# Transpulmonary thermodilution (TPTD) before, during and after Sustained Low Efficiency Dialysis (SLED). A Prospective Study on Feasibility of TPTD and Prediction of Successful Fluid Removal

**DOI:** 10.1371/journal.pone.0153430

**Published:** 2016-04-18

**Authors:** Wolfgang Huber, Stephan Fuchs, Andreas Minning, Claudius Küchle, Marlena Braun, Analena Beitz, Caroline Schultheiss, Sebastian Mair, Veit Phillip, Sebastian Schmid, Roland M. Schmid, Tobias Lahmer

**Affiliations:** 1 II. Medizinische Klinik und Poliklinik, Klinikum rechts der Isar; Technische Universität, München, München, Germany; 2 Klinik für Anaesthesiologie, Klinikum rechts der Isar der Technischen Universität München, München, Germany; Duke University Medical Center, UNITED STATES

## Abstract

**Background:**

Acute kidney injury (AKI) is common in critically ill patients. AKI requires renal replacement therapy (RRT) in up to 10% of patients. Particularly during connection and fluid removal, RRT frequently impairs haemodyamics which impedes recovery from AKI. Therefore, “acute” connection with prefilled tubing and prolonged periods of RRT including sustained low efficiency dialysis (SLED) has been suggested. Furthermore, advanced haemodynamic monitoring using trans-pulmonary thermodilution (TPTD) and pulse contour analysis (PCA) might help to define appropriate fluid removal goals.

**Objectives, Methods:**

Since data on TPTD to guide RRT are scarce, we investigated the capabilities of TPTD- and PCA-derived parameters to predict feasibility of fluid removal in 51 SLED-sessions (Genius; Fresenius, Germany; blood-flow 150mL/min) in 32 patients with PiCCO-monitoring (Pulsion Medical Systems, Germany). Furthermore, we sought to validate the reliability of TPTD during RRT and investigated the impact of “acute” connection and of disconnection with re-transfusion on haemodynamics. TPTDs were performed immediately before and after connection as well as disconnection.

**Results:**

Comparison of cardiac index derived from TPTD (CItd) and PCA (CIpc) before, during and after RRT did not give hints for confounding of TPTD by ongoing RRT. Connection to RRT did not result in relevant changes in haemodynamic parameters including CItd. However, disconnection with re-transfusion of the tubing volume resulted in significant increases in CItd, CIpc, CVP, global end-diastolic volume index GEDVI and cardiac power index CPI. Feasibility of the pre-defined ultrafiltration goal without increasing catecholamines by >10% (primary endpoint) was significantly predicted by baseline CPI (ROC-AUC 0.712; p = 0.010) and CItd (ROC-AUC 0.662; p = 0.049).

**Conclusions:**

TPTD is feasible during SLED. “Acute” connection does not substantially impair haemodynamics. Disconnection with re-transfusion increases preload, CI and CPI. The extent of these changes might be used as a “post-RRT volume change” to guide fluid removal during subsequent RRTs. CPI is the most useful marker to guide fluid removal by SLED.

## Introduction

Acute kidney injury (AKI) occurs in up to 30% of critically ill patients, requires renal replacement therapy (RRT) in up to 10% and markedly increases mortality [1–6]. Furthermore, AKI results in prolonged length of stay and additional costs as well as permanent renal failure and chronic renal replacement therapy (CRRT) in a substantial number of patients.

If RRT is required due to AKI, similar risks and side effects have to be considered as during chronic renal replacement therapy (CRRT). These risks include the need for anticoagulation, intra-dialytic hypotension as well as difficulties in defining and reaching dry weight [7]. However, with regard to critical illness, co-morbidities and greater instability in organ functions in general, circulatory side effects might be particularly difficult to predict and to manage. Haemodynamic instability due to RRT further impairs recovery of renal function [6,8,9]. To limit circulatory side effects, a number of approaches are used, including prolonged periods of RRT with reduced efficiency including sustained low efficiency dialysis (SLED) and continuous veno-venous haemo(dia)filtration (CVVH(D)F) [10].

Another approach is optimization of hemodynamics based on modern haemodynamic monitoring. The most recent guideline suggest using protocol-based management of haemodynamics and oxygenation parameters to prevent development or worsening of AKI in high-risk patients in the perioperative setting or in patients with septic shock [11].

Prediction of feasibility of fluid removal has been investigated in CRRT [7]. In addition to clinical examination this is usually based on less invasive methods including sonography (inferior vena cava diameter), echocardiography, bioimpedance and biochemical parameters including blood volume monitoring.

Depending on the underlying disease, a substantial number of critical care patients are equipped with advanced hemodynamic monitoring including pulse contour analysis (PCA) and trans-pulmonary thermodilution (TPTD). Both principles are combined in at least two commercially available devices (PiCCO-device; Pulsion Medical Systems SE, Feldkirchen, Germany; EV1000; Edwards Lifesciences, Irvine, USA). TPTD is used for indicator dilution to measure the static preload marker global end-diastolic volume index GEDVI [12–15], the pulmonary edema marker extravascular lung water index EVLWI [16,17] and cardiac index CI (CItd). PCA provides continuous assessment of CI (CIpc) after calibration by TPTD as well as dynamic parameters of fluid responsiveness such as stroke volume variation (SVV) and pulse pressure variation (PPV) which have been associated to outcome [18].

These parameters have been investigated extensively regarding prediction of “fluid responsiveness”, which is defined as a substantial increase in CI in response to a volume challenge. Moreover, these parameters might be useful to predict the feasibility and extent of fluid removal by RRT (“fluid removability”). Compared to the plethora of data on *fluid responsiveness* there is an amazing lack of data on *fluid removability* by RRT.

**Fig 1:**
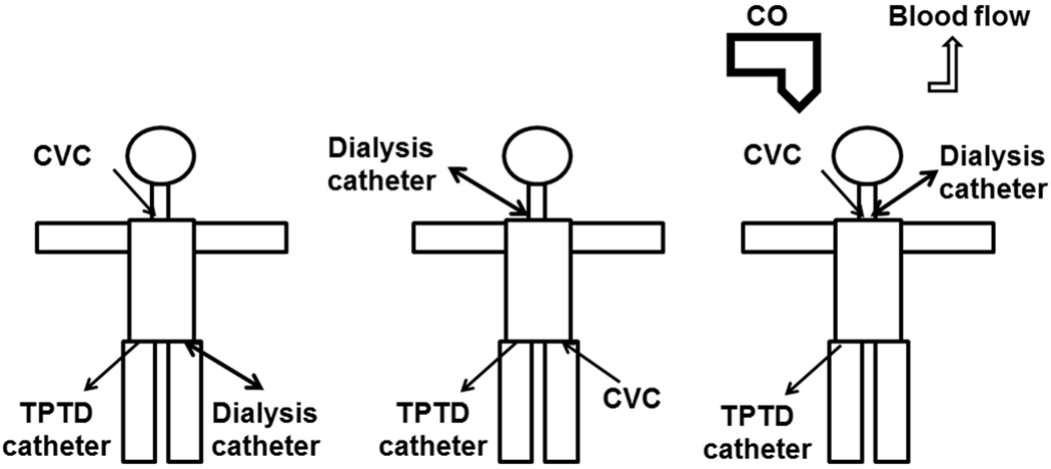
Potential impact of renal replacement therapy (RRT) on transpulmonary thermodilution (TPTD) with potential impact of catheter positions and blood flow.

This might be related to concerns on the applicability of indicator dilution techniques including TPTD during RRT. From a theoretical viewpoint several potential confounders of thermodilution have to be considered (Fig 1) including loss of indicator in the extracorporeal circuit, interference of temperature changes induced by the extracorporeal circuit and changes in blood pump flow. Furthermore, positioning of the CVC and dialysis catheter in the same position might impair TPTD (both catheters in jugular veins or both catheters in the femoral veins). Some of these interactions are difficult to differentiate from true side effects of RRT including a decrease in preload, cardiac output and arterial pressure as well as an increase in heart rate (HR) and changes in systemic vascular resistance index (SVRI).

**Fig 2:**
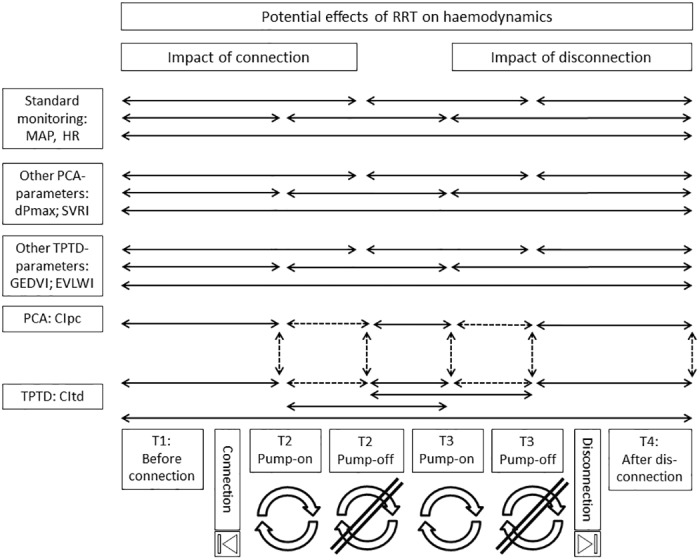
Flow-chart of potential comparisons before, during and after renal replacement therapy (RRT). Solid lines are used for comparisons aimed at potential real haemodynamic effects of RRT. Dashed lines are given for comparisons aimed at validation of transpulmonary thermodilution (TPTD) technique. MAP: mean arterial pressure. HR: heart rate. PCA: pulse contour analysis. GEDVI: global end-diastolic volume index. EVLWI: extra-vascular lung water index. CI: cardiac index.

To investigate potential confounding and impact of the RRT itself, haemodynamic measurements can be analyzed at a minimum of 6 different times (Table 1; Fig 2): before (T1) and after connection to RRT with (T2_on_) and without (T2_off_) blood flow induced by the blood pump as well as before disconnection with (T3_on_) and without (T3_off_) blood pump, and finally after disconnection (T4).

**Table 1:** Potential experimental settings.

Time	RRT-setting	Comparison	Purpose
T1	Before connection		Baseline without RRT
T2_on_	After connection and pump-on	T2_on_ vs. T1	Impact of connection
T2_off_	After connection and pump-off	T2_off_ vs. T2_on_	Impact of pump on TPTD-parameters
T3_on_	Before disconnection with pump-on	T3_on_ vs. T2_on_	Changes in TPTD induced by RRT
T3_off_	Before disconnection with pump-off	T3_off_ vs. T2_on_ Comparison vs. T3 _on_	Changes in TPTD induced by RRT;
T3_off_	Before disconnection with pump-off	Comparison vs. T3 _on_	Impact of pump on TPTD-parameters
T4	After disconnection	T4 vs. T1;	Changes in TPTD by RRT after disconnection
T4	After disconnection	T4 vs. T3	Impact of disconnection and re-transfusion of blood in the tubing

The number of potential study protocols can be multiplied by also considering different techniques of RRT (dialysis, SLED, CVVH(D)F) and the modality of connection which can be performed in an “acute” modality with pre-filled tubing and in a “chronic” RRT modality without pre-filling.

A small number of studies systematically investigated advanced haemodynamic monitoring during RRT in different settings (Table 2). These trials predominantly analyzed the impact of the pump-driven blood flow on TPTD-derived parameters (T2_on_ vs. T2_off_ and T3_on_ vs. T3_off_) and the overall impact of RRT on haemodynamics (T4 vs T1) [19–26]. Data are conflicting, since one study demonstrated significant alteration of CItd by ongoing CVVHF [22], while two other studies did not show a significant impact of blood-flow rates up to 350ml/min on TPTD-derived parameters [19,23].

**Table 2:** Studies investigating TPTD and PCA in RRT.

Author	RRT	Period of RRT	Blood flow [ml/min]	No. of patients	No. of RRTs	Investigated parameters	T1-T6	TPTD-Methodology endpoint investigated	Effect of fluid removal investigated	Effect of retransfusion investigated
Kuhn 2006 [20]	Dialysis (CRRT)	4h		42	42	GEDVI; EVLWI	T1, T4	-	+	-
Sakka 2007 [19]	CVVHF	Not given	80–150	24	24	CI; ITBVI; EVLWI	T1, T2_on_, T2_off_, T4	+	+	-
Compton 2007 [21]	Dialysis ARF	4h	Not given	9	39	CI; ITBVI; EVLWI	T1, T4	-	+	-
Heise 2012 [22]	CVVHF	Not given	183±35	32	32	CO	T2_on_, T2_off_,	+	-	-
Dufour 2012 [23]	CVVHF	Not given	250–350	69	69	CI; GEDVI; EVLWI	T2_on_, T2_off_	+	-	-
De Laet 2012 [25]	CVVH, SLED	6h (SLED)	150–300	9	25	IAP; GEDVI; EVLWI	T1, T4	-	+	-
Pathil 2012 [24]	SLED	8h	Not given	30	30	CI; GEDVI; EVLWI	T1, T2_on_	+	-	-
Compton 2014 [26]	Dialysis	4h	Not given	35	35	CI; ITBVI; EVLWI	T1, T4	-	+	-
Own study	SLED	10h	150	32	51	CI; GEDVI; EVLWI; CPI	T1, T2_on_, T3_on_, T4	+	+	+

TPTD: transpulmonary thermodilution; PCA: pulse contour analysis; RRT: renal replacement therapy

In addition to these conflicting data, to the best of our knowledge there is limited or no data available on the impact of connection to RRT in “acute modality” with pre-filled tubing (T1 vs. T2_on_) and on the impact of re-transfusion at the end of RRT (T3_on_ vs. T4). Finally, there is a lack of data on the prediction of the feasibility of fluid-removal during SLED by modern haemodynamic parameters.

### Aims of the study

1. Therefore, in a first step we investigated *technical issues* including the validity of TPTD-derived parameters during SLED by comparing TPTD-derived CItd (which could be potentially confounded by loss of indicator) before and after (dis)connection of SLED to CIpc which should be independent of loss of indicator,
2. In a second step we analyzed feasibility and impact of fluid changes during RRT including
  - the impact of *“iso-volaemic acute”* connection to RRT with pre-filled tubing,
  - the impact of *“hypervolaemic”* disconnection with re-transfusion of the blood within the tubing within 2–3 minutes (“post-RRT-volume challenge”) and
  - the predictive capabilities of haemodynamic parameters before and in the course of RRT regarding “volume removability” of the pre-defined filtration-goal.

Based on this approach we aimed to investigate feasibility and usefulness of TPTD before and during SLED. Overall, the aims of the study could be achieved.

## Materials and Methods

The study was approved by the institutional review board (Ethikkommission Technische Universität München; Fakultät für Medizin; No. 3049/11). We analyzed data of a prospectively maintained anonymized and de-identified haemodynamic database including 208 TPTD measurements before, during and after 52 SLED therapies in 31 consecutive patients treated in a general ICU. All patients were under haemodynamic monitoring with the PiCCO-2-device (Pulsion Medical Systems SE, Feldkirchen, Germany) and on RRT irrespective of the study. The need for informed consent was waived due to the non-interventional design of the study.

The patients characteristics are shown in Table 3.

**Table 3:** Patients characteristics.

**Based on individual patients (n = 32)**	
Age	64±9
Gender [years]	22 male; 10 female
Height [cm]	172±7
Weight [kg]	78±23
APACHE-II	25±8
Aetiology	
- Sepsis	11/32 (34.4%)
- ARDS	8/32 (25%)
- Liver failure	7/32 (21.9%)
- Gastrointestinal bleeding	4/32 (12.5%)
- Cardiogenic shock	1/32 (3.1%)
- CNS affection	1/32 (3.1%)
**Based on RRT-sessions (n = 51)**	
Venous access	
- CVC jugular, dialysis catheter femoral	43/51 (84.3%)
- CVC subclavian, dialysis catheter femoral	1/51 (2.0%)
- CVC femoral, dialysis catheter jugular	7/51 (13.7%)
Anticoagulation	
- Heparin	22/51 (43.1%)
- Citrate	19/51 (37.3%)
- Argatroban	2/51 (3.9%)
- Without anticoagulation	8/51 (15.7%)
Catecholamines at baseline	29/51 (56.9%)
Ultrafiltration-goal [mL]	1527±891
Ultrafiltration achieved [mL]	1424±975
Blood-flow [ml/min]	148.8±13.6

For each SLED treatment the dataset consisted of four triplicate TPTDs with 15 mL of iced saline. TPTDs were performed as described previously [13] immediately before (T1) and 5 min after (T2) connection to SLED as well as immediately before (T3) and after (T4) disconnection of SLED. Immediately before each TPTD, PCA-derived parameters, RR and heart rate were documented.

The cardiac power index CPI was calculated as the product of mean arterial pressure [mmHg] and cardiac index [L/min/m^2^] divided by 451 [27].

Measurements at T2 and T3 during RRT were performed without reducing the flow of blood and dialysate. Therefore, in the context of this study we used “T2” and “T3” instead of “T2_on_”and “T3_on_“.

SLED was performed using the GENIUS 90 device (Fresenius Medical Care Deutschland GmbH; Bad Homburg v.d.H., Germany) with FX40, FX50 or FX60 dialyzers containing 32 mL, 53 mL and 74 mL, respectively. Dialyzers were connected with GENIUS 90 XS tubing (Fresenius Medical Care Deutschland GmbH; Bad Homburg v.d.H., Germany) with a volume of 121 mL. FX40, FX50 or FX60 dialyzers and tubing were pre-filled with a total of 153 mL, 174 mL or 195 mL of 0.9% saline, respectively. Blood and dialysate flow were set at 150 mL/min.

For the venous access for RRT Gambro Gam Cath Dolphin-catheters were used. Catheters with a length of 250mm and a diameter of 13 F were used for femoral access and catheters with a length of 150–175 mm and a diameter of 13 F were used for jugular RRT-access, respectively. CVC and dialysis catheters were inserted in different positions (one in vena cava superior and the other one in vena cava inferior).

### Endpoints

In chronological order the following endpoints were investigated:

1. Comparison of CIpc-T2 vs. CItd-T2 to analyze validity of CItd-T2 which could be confounded by loss of indicator during ongoing RRT, which does not apply to CIpc-T2 recorded immediately before TPTD-T2 [23].
2. Comparison of changes in CItd vs. changes in CIpc before and after connection to RRT (CItd-T2 –CItd-T1 vs. CIpc-T2 –CIpc-T1) to analyze the validity of changes in CItd after initiation of RRT which could be confounded by loss of indicator during ongoing RRT, which does not apply to changes in CIpc.
3. Comparisons of CItd-T2 to CItd-T1 and of CIpc-T2 to CItd-T1 to investigate the impact of acute connection to RRT on haemodynamics with pre-filled tubing.
4. To further investigate the impact of *“iso-volaemic”* connection to RRT on haemodynamics we compared parameters derived from TPTD (EVLWI, GEDVI) as well as MAP, CVP, systemic vascular resistance index (SVRI) and heart rate before and after connection to RRT (T2 vs. T1).
5. Comparison of CIpc-T4 vs. CItd-T4 to analyze validity of CItd-T4 which could be confounded by loss of indicator during ongoing RRT, which does not apply to CIpc-T4 recorded immediately before TPTD-T3.
6. Comparison of changes in CItd vs. changes in CIpc before and after disconnection to RRT (CItd-T4 –CItd-T3 vs. CIpc-T4 –CItd-T3) to analyze validity of changes in CItd after termination of RRT which could be confounded by loss of indicator during ongoing RRT, which does not apply to changes in CIpc.
7. Comparisons of CItd-T4 to CItd-T3 and of CIpc-T4 to CItd-T3 to investigate the impact of a”*hyper-volaemic”* disconnection to RRT with re-transfusion of the blood within the tubing within 2–3 minutes (“post-RRT-volume challenge”) on haemodynamics with pre-filled tubing.
8. To further investigate the impact of”*hyper-volaemic”* disconnection to RRT on haemodynamics we compared parameters derived from TPTD (EVLWI, GEDVI) as well as MAP, CVP, SVRI and heart rate HR before and after disconnection to RRT (T4 vs. T3).
9. The **primary endpoint** was the analysis of the predictive capabilities of haemodynamic parameters regarding “fluid removability” which was defined as achieving ≥90% of the pre-defined net-filtration goal without an increase in the vasopressor dosage of more than 10% or initiation of a vasopressor. The net-filtration goal was defined by an investigator not directly involved in the study.
  In a first step we analyzed predictive capabilities of baseline haemodynamic parameters including MAP, CVP, CI, SVI, GEDVI, EVLWI and CPI.
10. In a second step we analyzed predictive capabilities of changes in continuously (MAP, CIpc, heart rate, CPI) and discontinuously measured parameters in course of RRT compared to baseline.

### Statistical analyses

To compare changes in continuous variables before and after connection to RRT we used Wilcoxon-test for paired samples. To compare continuous parameters between different groups achieving and failing the primary endpoint, the Wilcoxon-test for unpaired samples was used.

Multivariate binary regression analysis regarding the primary endpoint was performed including variables which were significantly different between patients achieving or failing the primary endpoint or with a p-value <0.2 in univariate analysis.

Receiver operating characteristics (ROC)-analyses were performed to assess discriminative ability of univariate predictor variables regarding the primary endpoint.

In a limited number of TPTD measurements, single haemodynamic variables could not be obtained. Therefore, statistical tests were calculated based on the measurements with valid data.

All statistical tests were performed on a two-sided level of significance of α = 5% using IBM SPSS Statistics 23 (SPSS Inc., Chicago, IL, USA).

## Results

### Validity of TPTD after connection and disconnection to dialysis RRT

Hypothesizing that TPTD, but not PCA might be confounded by the RRT-blood pump we compared CItd during RRT to CIpc recorded immediately after connection to RRT (CItd-T2 vs. CIpc-T2) and after disconnection connection from RRT (CItd-T4 vs. CIpc-T4).

CItd-T2 was not significantly different to CIpc-T2 (4.29±1.48 vs. 4.25±1.38 L/min/m^2^; p = 0.983). Furthermore, changes in CItd (CItd-T2 –CItd-T1) and changes in CIpc (CIpc-T2 –CItd-T1) after connection were not significantly different (-0.12±0.56 vs. -0.15±0.50; p = 0.983).

After disconnection CItd-T4 was slightly different to CIpc-T4 (4.18±1.43 vs. 4.08±1.48 L/min/m^2^; p = 0.047). Similarly, changes in CItd (CItd-T4 –CItd-T3) after disconnection were slightly higher than changes in CIpc (CIpc-T4 –CItd-T3; 0.28±0.35 vs. 0.19±0.37; p = 0.047). Regarding the small differences and borderline levels of significance, this can be considered as clinically not relevant.

### Impact of isovolaemic connection to RRT

**Fig 3:**
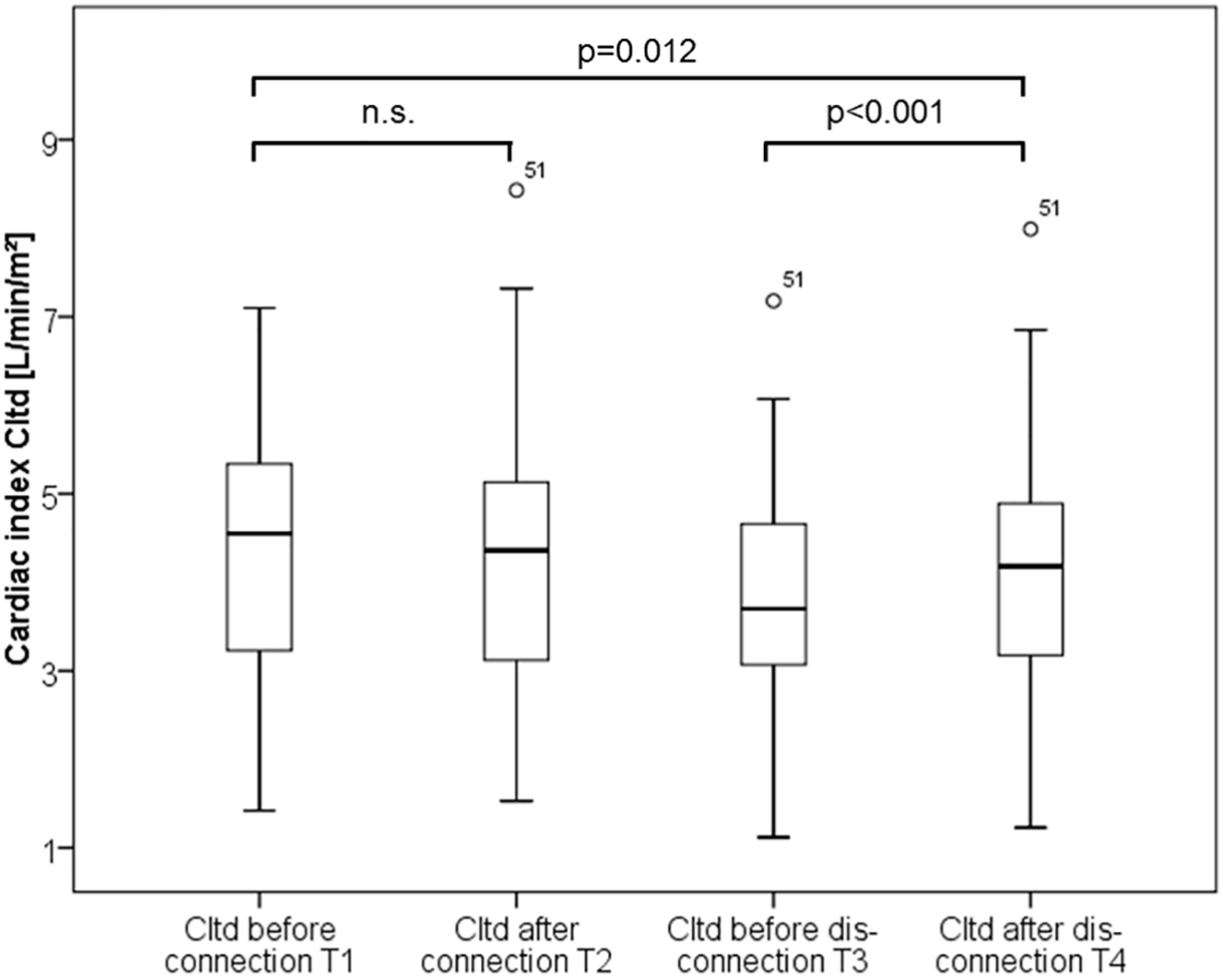
Boxplots comparing thermodilution derived cardiac index (CItd) values over time.

**Fig 4:**
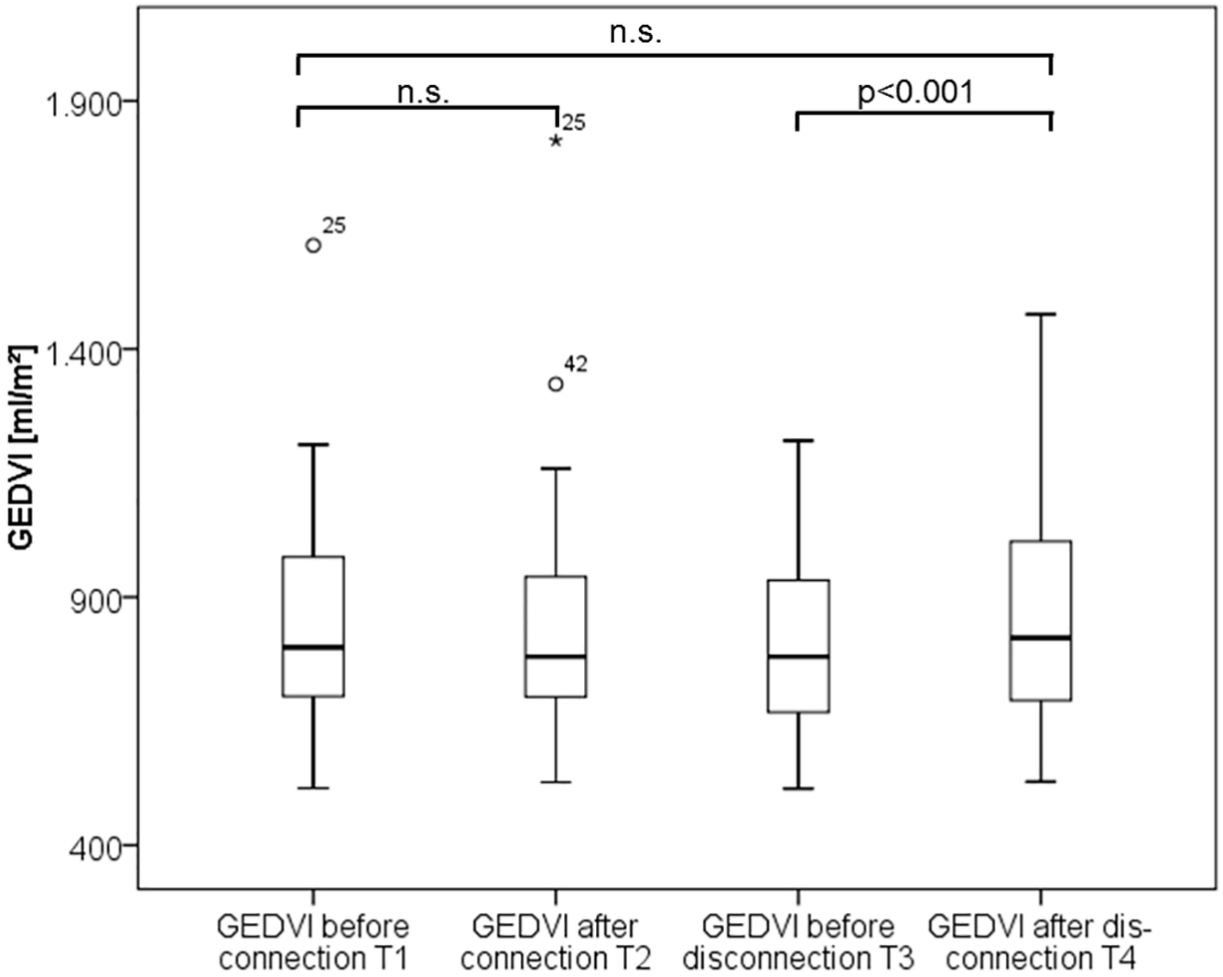
Boxplots comparing global end-diastolic volume index (GEDVI) values over time.

**Fig 5:**
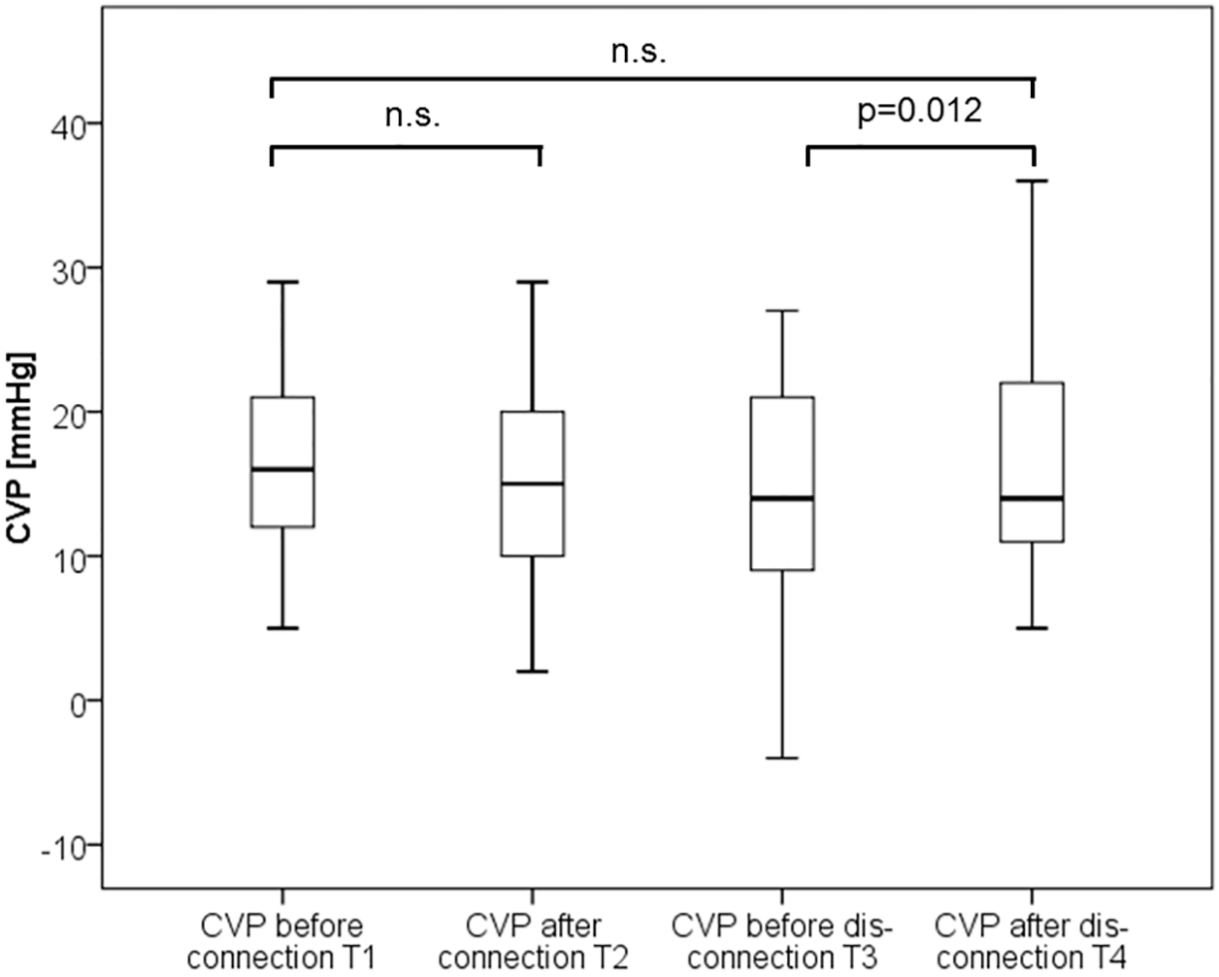
Boxplots comparing central venous pressure (CVP) values over time.

Isovolaemic connection to RRT did not result in any significant changes in CItd, CIpc, CVP, GEDVI, EVLWI, MAP, heart rate and dPmax (Table 4; Figs 3–5). Only CPI marginally decreased (0.77±0.32 vs. 0.78±0.27; p = 0.037). In summary these data suggest no substantial impact of isovolaemic connection to SLED on haemodynamics.

**Table 4:** Haemodynamic parameters over time.

	T1	T2	p-value T2 vs. T1	T3	T4	p-valueT4 vs. T3	p-value T4 vs. T1
CItd [L/min/m^2^]	4.41±1.41	4.29±1.48	p = 0.063	3.90±1.31	4.18±1.43	p<0.001	p = 0.012
CIpc [L/min/m^2^]	4.41±1.41*	4.25±1.38	p = 0.068	3.90±1.31	4.08±1.48	p = 0.004**	p = 0.001
GEDVI [mL/m^2^]	847±206	845±227	p = 0.574	813±182	860±215	p<0.001	p = 0.095
EVLWI [ml/kg	11.5±4.8	11.6±5.1	p = 0.938	11.2±5.7	10.9±4.4	p = 0.579	p = 0.102
CVP [mmHg]	16.5±6.7	15.8±7.2	p = 0.352	14.8±7.5	15.9±7.9	p = 0.012	p = 0.692
Heart rate [min^-1^]	95±15	95±14	p = 0.562	91±19	89±22	p = 0.168	p = 0.005
MAP [mmHg]	80.6±13.9	80.6±13.0	p = 0.201	84.0±12.4	84.5±12.3	p = 0.923	p = 0.044
dPmax [mmHg/s]	1615±621	1603±637	p = 0.360	1769±690	1761±760	p = 0.952	p = 0.295
CPI [W/m^2^]	0.78±0.27	0.77±0.32	p = 0.037	0.73±0.29	0.78±0.31	p = 0.003	p = 0.793

* CIpc-T1 = CItd-T1 per definition

** CIpc-T4 was compared to CItd-T3 which is CIpc immediately after TPTD per definition

All other CIpc-values were analyzed as documented immediately before the subsequent TPTD

### Impact of hypervolaemic disconnection

By contrast, hyper-volaemic disconnection to RRT with re-transfusion of the blood within the tubing within about two minutes resulted in significant increases in the preload markers GEDVI and CVP as well as CIpc, CItd and CPI, suggesting a certain kind of “volume response” of preload and CI to re-transfusion (Table 4; Figs 3–5).

By contrast EVLWI, MAP and dPmax were not significantly different after disconnection.

### Prediction of fluid removability

The net ultrafiltration goal defined before the initiation of RRT was 1527±891ml, while net-ultrafiltration achieved after 619±70 minutes of SLED was 1424±975ml. Total ultrafiltration was 1942±1174ml. Blood flow was 147±14ml/min.

In 22 of 51 RRT treatments the primary endpoint (at least 90% of the net-filtration goal achieved without increase in catecholamines) was fulfilled. In 18 of 51 RRTs (35%) less than 90% of the pre-set goal could be filtrated, in 16 of 51 RRTs the catecholamine dosage had to be increased. In five RRTs less than 90% of the filtration goal could be achieved despite increase in the catecholamine dosage.

Patients fulfilling the primary endpoint had significantly higher baseline values of CPI (0.90±0.27 vs. 0.69±0.23 W/m^2^; p = 0.016) and CI (4.98±1.34 vs. 4.07±1.48 L/min/m^2^) compared to patients not fulfilling the primary endpoint.

All other baseline parameters including CVP, GEDVI, SVI, dPmax, EVLWI, HR, SVRI, S_cv_O_2_, noradrenaline-dosage, age and APACHE-II were not predictive regarding the primary endpoint.

In multivariate analysis also including baseline values of CPI, CVP, GEDVI, CI, SVI, MAP, S_cv_O_2_, noradrenaline dosage and the net-filtration goal, CPI was the only parameter independently associated with the primary endpoint (p = 0.011).

**Fig 6:**
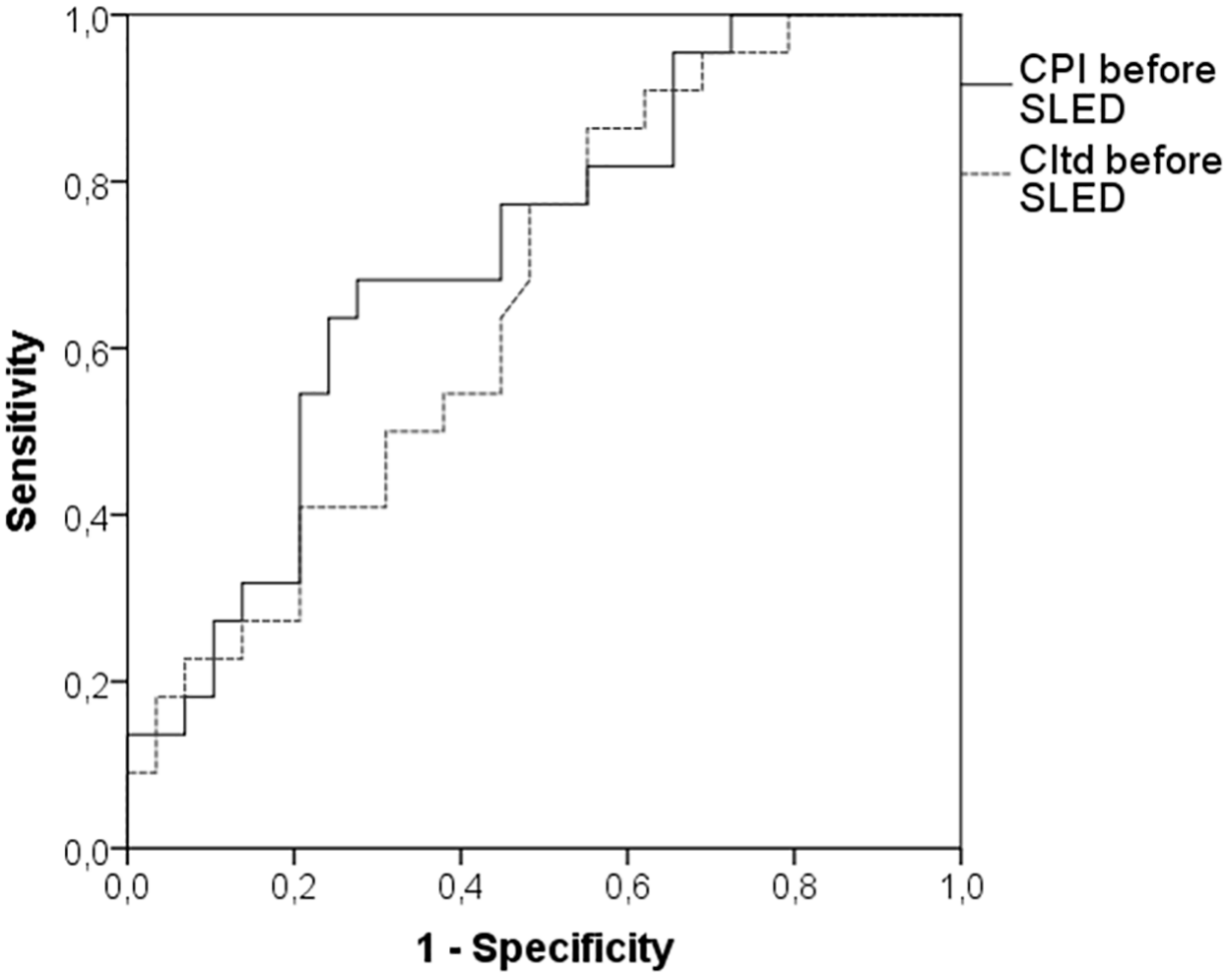
ROC-analysis regarding the primary endpoint.

The ROC-AUC regarding the primary endpoint was 0.712 (95%-CI: 0.570–0.853; p = 0.010) for baseline CPI and 0.662 (95%-CI: 0.514–0.810; p = 0.049) for baseline CI (Fig 6). A cut-off of 0.72 W/m^2^ for CPI had a sensitivity of 77% and a specificity of 55% regarding the primary endpoint.

Among changes in continuously reported haemodynamic parameters after connection and before disconnection only changes in CPI compared to CPI after connection were significantly associated to the primary endpoint: The difference between PCA-derived CPI before disconnection and CPI after connection (CPIpc-T3 –CPItd-T2) was 0.07±0.18 W/m^2^ in patients fulfilling the primary endpoint compared to -0.0431±0.18 W/m^2^ (p = 0.029) in those not fulfilling the primary endpoint.

### Overall changes in hemodynamic parameters

Comparison of values after disconnection to values before connection demonstrates slight decreases in CItd (4.18±1.43 vs. 4.41±1.41 L/min/m^2^; p = 0.012) and heart rate (89±22 vs. 95±15/min; p = 0.005), but a moderate increase in MAP (84.5±12.3 vs. 80.6±13.9 mmHg; p = 0.044) and noradrenalin dosage (1.18±2.01 vs. 0.88±1.63 mg/h; p = 0.038).

By contrast CPI (0.80±0.34 vs. 0.78±0.27; p = 0.793), GEDVI, EVLWI and dPmax were not changed by RRT (Table 4).

## Discussion

AKI requiring RRT is associated with impaired long-term outcome and mortality. Therefore, avoidance of AKI and limitation of side effects of RRT are major goals of intensive care. RRT itself is associated with potential risks including complications of anti-coagulation and cardiovascular side effects which can also impair recovery of AKI. Modern haemodynamic monitoring and optimization of fluid balance hold the potential of reducing the risks of AKI and RRT.

Nevertheless, there is a lack of data on the use of TPTD and PCA in patients with AKI and RRT. This might, in part, be related to concerns about confounding of TPTD-results by RRT itself. While numerous studies investigate prediction of fluid responsiveness, there is scarce data on the use of TPTD and PCA to predict feasibility of fluid removal.

Therefore, our study was elaborately designed to investigate potential confounding of TPTD by RRT, the impact of connection and disconnection of SLED on haemodynamics and finally the prediction of “fluid removability” in ICU patients with RRT.

The main results can be summarized as follows:

1. Comparison of absolute values and changes in CItd to CIpc before and after (dis)connection did not give any hints for a substantial impairment of TPTD by SLED. Although CItd-T4 and CIpc-T4 were significantly different, the bias of -0.097±0.31 L/min/m^2^ and a percentage error of 14.7% would be in line with an “interchangeability” of CItd and CIpc.
2. Connection to SLED in an “acute modality” with pre-filled tubing did not result in haemodynamic instabilities or in any significant changes of haemodynamic parameters derived from TPTD, PCA or routine measurements at all.
3. By contrast, disconnection with re-transfusion of the blood contained in the tubing induced an increase in preload, cardiac output and MAP. Therefore, re-transfusion can be considered as a kind of volume challenge at the end of SLED and filtration.
4. Finally, our data demonstrate that higher baseline values of CPI and CI are predictive for achieving pre-set goals for ultrafiltration without substantial increase in the vasopressor dosage.

With regard to the trial design, in a first step we investigated the validity of CItd during ongoing RRT. Our results give no hints of confounding of CItd by low blood-flow RRT which is in line with the majority of the limited data available [19,22,23]. There are three approaches to investigate the impact of blood flow on TPTD-derived parameters in a clinical routine setting:

1. Comparison of CItd (and other parameters derived from TPTD) to CItd with pump on or off.
  This approach was investigated by at least three trials comparing three consecutive CItd measurements with pump_on_, pump_poff_ and pump_on_ setting [19,22,23]. As summarized by Sakka et al., the studies performed during CVVHF demonstrated that “RRT had no clinically relevant effect on measurement of CI” [19]. Only one study analyzing 15 single TPTDs (each five before, during and after stopping the blood-pump) in 32 ICU patients demonstrated that only the first of several measurements after changing the blood-flow might be confounded [22]]. Therefore, the authors concluded that interruption of CRRT before measuring CO is not recommended [22]. Since these data suggest that stopping the blood-flow confounds TPTD, and also considering clinical routine, our study design did not include a stop of the blood-pump.
2. Comparison of CItd to CIpc during and after stop of blood-flow.
  Assuming that CIpc is not confounded by RRT, but CItd might be confounded by RRT, potential differences between CItd and CIpc during ongoing blood-flow must be related to confounding TPTD by RRT blood-flow. This approach was used by Dufour et al. and did not show any confounding of CItd by a blood-flow up to 350ml/min [23].
3. Comparison of CItd to CIpc with ongoing blood-flow.

Since in clinical routine a stop of the RRT-pump for haemodynamic measurements might be cumbersome, we compared TPTD-derived CItd to CIpc with ongoing pump. Similar to the previous studies our data confirm that CItd can be accurately measured during RRT.

Stability of EVLWI and GEDVI after iso-volaemic “acute” connection to RRT in our study suggests that also these parameters are not substantially confounded by ongoing RRT.

In general, the *effects of iso-volaemic “acute” connection* to RRT have not been extensively investigated. In patients with CRF and CRRT a fluid-overload at the onset of RRT is generally assumed. Therefore, patients undergoing chronic RRT are usually connected in a “chronic” modality without pre-filling of the tubing. By contrast, in acute RRT, in particular in critical care patients with high risk of haemodynamic instability, connection to the RRT is established in an “acute” modality, with the tubing being pre-filling. To the best of our knowledge, there are no data using advanced haemodynamic monitoring to investigate the impact of acute modality of connection to RRT in ICU-patients. Our data suggest that connection to RRT in an acute modality does not impair haemodynamic parameters derived from standard monitoring, TPTD or PCA.

While connection to RRT in ARF is aimed at avoidance of circulatory impairment, disconnection usually is performed in a re-transfusion technique to avoid a loss of blood contained within the tubing. In our unit re-transfusion is performed with a blood-flow of 60–100 mL/min resulting in a re-transfusion time of 1.5 to 4 minutes depending also on the volume of the dialyzer used. Several recent papers suggest the usefulness of “mini volume challenges” using fluid boluses of 100 mL infused over one minute [28,29]. While these volume challenges are used irrespective of RRT as an additional test to assess fluid responsiveness in case of suspected hypovolaemia, re-transfusion at disconnection could have the potential for a “post-RRT re-transfusion volume-challenge” (PRRVC). This PRRVC might predict, if fluid removal was appropriate or resulted in critical volume depletion.

Despite these promising findings, PRRVC has to be considered as an ex-post analysis of fluid removal. Therefore, we chose the a priori prediction of fluid removability as the primary endpoint. In this study the ultrafiltration goal defined immediately before RRT was not associated to the primary endpoint. This indicates that not the amount of ultrafiltration per se, but the individual patient´s condition was decisive for the primary endpoint. Interestingly, two parameters derived from TPTD (CItd; CPItd) were the only significant predictors of feasibility of the pre-defined ultrafiltration goal. The impact of lower values of CItd is remarkable regarding a mean CItd of 4.41±1.41 L/min/m^2^ and suggests that haemodynamic stability of patients “normal”CI might be overestimated, in particular in a setting with a large proportion of septic patients requiring noradrenalin to keep MAP above 65 mmHg. This might also explain the significant predictive role of CPI, which combines CI and MAP in one parameter. This parameter–whether indexed as CPI or unindexed and termed Cardiac Power Output CPO—has already been demonstrated as the strongest haemodynamic correlate of mortality in cardiogenic shock more than a decade ago [27]. Despite further promising data [30,31], CPI is not routinely used in the setting of RRT. Based on simple multiplication of CI, MAP and a constant, CPI can be determined with a variety of devices with the minimum requirement of accurate measurement of CI. As mentioned in the methods section we calculated CPI and did not use the one-digit display provided by the PiCCO-device. Regarding the prognostic potential of this parameter, we would suggest optimization of the display of the PiCCO-device, since otherwise important information might get lost. E.g. a CPI displayed as 0.3 W/m^2^ might be 0.25 W/m^2^ or 0.34 W/m^2^ which is a difference of 36%. With regard to the combined approach of TPTD and PCA in our study, also the association of changes over time in PCA-determined CPI to failure in fluid removal is of interest. Nevertheless, with a ROC-AUC of 0.712, a sensitivity of 77% and a specificity of 55% the predictive capabilities of CPI at baseline have to be classified as moderate.

### Practical applications, strengths and limitations of the study

Overall, this study confirms feasibility and usefulness of TPTD during RRT. Using a different setting compared to previous studies, our data demonstrate that TPTD is accurate despite ongoing RRT. Furthermore, based on advanced haemodynamic monitoring this study shows that “acute iso-volaemic connection” avoids haemodynamic impairment during connection of the patient to the RRT. Additionally, an increase in preload, contractility and markers of general cardiac performance by hypervolaemic disconnection demonstrate a potential for a “post-RRT re-transfusion volume-challenge” (PRRVC) to guide ultrafiltration goals for subsequent RRTs.

Baseline CPI and its changes during RRT are promising parameters to guide fluid removal during RRT.

Nevertheless, our study has several limitations: It was a single center study with a limited number of patients and repeated measurements in single patients. Our study was performed using a low blood flow of 150 mL/min, and with CVC and dialysis catheters strictly in different positions (one in vena cava superior and the other one in vena cava inferior). Therefore, it is not known, if our findings can be transferred to other RRT settings and catheter positions. In particular with regard to high-flow extracorporeal organ support such as extracorporeal membrane oxygenation (ECMO) our data has to be interpreted with caution.

Furthermore, we could not include dynamic indices of fluid responsiveness such as stroke volume variation (SVV) and pulse pressure variation (PPV) in our analysis. Several studies in the peri-operative setting demonstrated superior prediction of fluid responsiveness by SVV and PPV compared to filling pressures and end-diastolic volumes, However, the applicability of these parameters is low in the ICU-setting due to the frequent lack of sinus rhythm and controlled mechanical ventilation, which are mandatory criteria for the use of SVV and PPV [32–35]. These limitations also apply to our patients: The analysis of the 51 baseline measurements showed that both criteria mandatory (i.e. sinus rhythm and controlled mechanical ventilation) for the use of SVV or PPV were found in only 4 out of 51 datasets (7.8%).

Overall, haemodynamic changes induced by SLED with an ultrafiltration of about 1.5 L were—at best—moderate and did not result in substantial decreases in mean preload parameters, MAP and CI over time. However, it has to be kept in mind that these patients were on haemodynamic monitoring *before* inclusion in the study. Previous TPTD measurements might have been useful to balance preload, MAP and CI. Therefore, ultrafiltration goals and achieved rates of about 1.5 L were moderate and not aimed at massive de-resuscitation.

### Conclusions

TPTD is feasible during SLED. “Acute” connection does not substantially impair haemodynamics. Disconnection with re-transfusion increases preload, CI and CPI. The extent of these changes might be used as a “post-RRT volume change” to guide fluid removal during subsequent RRTs.

CPI is the most useful marker to guide fluid removal by SLED.
